# Parliament and the Profession

**Published:** 1917-12-01

**Authors:** 


### Parliament and the Profession.

### The Fire at Crumpsall.

The President of the Local Government Board stated that on learning, with deep regret, of the disastrous fire at the Delaunays Road Hospital of the Manchester Guardians, he immediately instructed the general inspector for the district to visit the institution and attend the inquest. Mr. Hayes Fisher added that he had called for reports as to the use of any temporary buildings of this nature in Poor-Law institutions, with a view to securing that adequate precautions are taken against risk of fire.

### Prisoners Certified Insane.

In answer to a question put by Mr. E. Harvey the Home Secretary stated that the Prison Commissioners are satisfied that the proportion of prisoners who are certified insane during imprisonment is not excessive, having regard to the classes from whence they are drawn, and the conditions of life under which they existed prior to conviction. They have no reason to think that the insanity is the result of imprisonment; but the close expert medical supervision in prison sometimes establishes the fact of insanity in cases where the symptoms would not be observed by medical men while the prisoner was at liberty.

### Doctors' Fees for Post-Mortem Examinations.

Mr. Ingleby asked the Home Secretary if he was aware that, in cases where a patient is brought into hospital in a moribund condition and a *post-mortem* examination has to be made and an inquest held, the medical officer who undertakes the *post-mortem* and subsequently attends the inquest to give evidence is precluded by Section 2, Clause 22, of Part II. of the Coroners Act, 1887, from receiving any fee, whereas if the patient is dead before being received into hospital the medical officer is, under Section 1, entitled to receive £2 2s. for his services; if he was aware that coroners have from time to time expressed their opinion in Court of the injustice suffered by the medical profession in this matter; and whether he would introduce a short Bill respecting the section of the Act. Sir George Cave said he believed that the facts were as stated in the question, but he could not see his way to propose legislation on the subject at present.

